# Genetic and pathological analysis of hooded cranes (*Grus monacha*) naturally infected with clade 2.3.4.4b highly pathogenic avian influenza H5N1 virus in South Korea in the winter of 2022

**DOI:** 10.3389/fvets.2024.1499440

**Published:** 2024-11-06

**Authors:** Ye-Ram Seo, Sun-Hak Lee, Sol Jeong, Hyunjun Cho, Daehun Kim, Dong-Ju Kim, Young-Jae Si, Hyesung Jeong, Suwoong Lee, Chang-Seon Song, David E. Swayne, Dong-Hun Lee

**Affiliations:** ^1^Avian Disease Laboratory, College of Veterinary Medicine, Konkuk University, Seoul, Republic of Korea; ^2^Wildlife Disease Research Team, National Institute of Wildlife Disease Control and Prevention, Gwangju, Republic of Korea; ^3^Zoonotic Disease Research Center, Konkuk University, Seoul, Republic of Korea; ^4^Birdflu Veterinarian LLC, Watkinsville, GA, United States; ^5^Wildlife Health Laboratory, College of Veterinary Medicine, Konkuk University, Seoul, Republic of Korea

**Keywords:** highly pathogenic avian influenza virus, H5N1, clade 2.3.4.4b, South Korea, wild bird, hooded crane

## 1 Introduction

Highly pathogenic avian influenza (HPAI) subtype H5Nx viruses of the A/Goose/Guangdong/1/1996 (Gs/Gd) lineage have caused substantial economic losses in the poultry industry and represent a significant public health concern (1). Since its first detection in 1996, it has diverged into 10 genetically distinct hemagglutinin (HA) clades (0–9) and subclades (2). Of these, the clade 2.3.4.4b HPAI H5N1 viruses have caused outbreaks in wild birds, poultry, and mammals in broad geographical regions including Asia, Europe, Africa, North America, South America, and Antarctica since 2020 (3–5). In particular, wild aquatic birds have played a key role in the maintenance and global spread of clade 2.3.4.4b HPAI viruses (6–8).

In South Korea, the clade 2.3.4.4b HPAI H5N1 viruses caused multiple outbreaks in wild birds, including 67 reported cases from October 2021 to March 2022, and 174 reported cases from October 2022 to March 2023 (9, 10). During October 2022-March 2023, the HPAI H5N1 virus was detected in 22 wild bird species (11). Particularly, there was a mass die-off event of 221 hooded cranes (*Grus monacha*) in Suncheon Bay, South Korea during November–December 2022. We conducted whole genome sequencing and comparative phylogenetic analysis of the isolates from hooded cranes. To elucidate the histopathological changes induced by clade 2.3.4.4b H5N1 HPAI viruses in hooded cranes, histopathological evaluation and immunohistochemistry were conducted on a hooded crane (sample no. 22WC-042) found dead in Suncheon Bay during November 2022 (Supplementary Figure S1).

## 2 Methods

### 2.1 Virus isolation and genome sequencing

Unprecedentedly, 221 hooded cranes (*Grus monacha*) were found dead in Suncheon Bay, South Korea during November–December 2022. The National Institute of Wildlife Disease Control and Prevention (NIWDC) of South Korea conducted virus isolation and collected internal organs from 211 carcasses to investigate the viral distribution. Oral and cloacal swab samples obtained from birds were placed in phosphate-buffered saline (PBS) containing 400 mg/mL antibiotic-antimycotic (Gibco, Grand Island, NY, USA) and thoroughly homogenized by vortexing for 1 min. For the isolation and identification of the virus, supernatant of swab samples was filtered using a 0.45 μm Syringe Filter. Filtered samples were inoculated into the allantoic cavities of 9–11-day-old specific pathogen-free embryonated eggs. After incubation for 72 h at 37°C, allantoic fluid was collected, and RNA extraction was performed using the Maxwell^®^ RSC simply RNA Tissue Kit (Promega, Madison, WI, USA) following the manufacturer's instructions. Subsequently, the samples were screened with the matrix (M) and H5 genes using real-time reverse transcription-PCR (rRT-PCR) (12). Complementary DNA synthesis was carried out using the SuperScript III First-Strand Synthesis system (Invitrogen, Carlsbad, CA, USA), followed by PCR targeting the hemagglutinin (HA) and neuraminidase (NA) genes to determine pathogenicity and subtypes (13). Among the 194 H5N1 HPAI virus-positive samples, 15 samples representing each outbreak week underwent next-generation sequencing (NGS) as previously described (14).

### 2.2 Phylogenetic analysis

The viral strains with the highest similarity to isolated H5N1 HPAIVs were identified using Basic Local Alignment Search Tool (BLAST) of the Global Initiative on Sharing all Avian Influenza Data (GISAID) database. For phylogenetic analysis, reference sequences were obtained from GISAID database. To select reference sequences, representative sequences were chosen by extracting clusters containing genome sequences of viruses from hooded cranes, and the ElimDupes program was used to exclude sequences showing more than 99.8% similarity (https://www.hiv.lanl.gov/content/sequence/elimdupesv2/elimdupes.html). The reference sequences and sequences isolated from the hooded crane were aligned using MAFFT (https://mafft.cbrc.jp/alignment/software/). Maximum-likelihood (ML) trees for 8 genes were constructed using RAxML v8.0 (15) with general time-reversible + Gamma model, 1,000 bootstrap replicates. Genotypes were determined based on criteria from a previous study (11).

### 2.3 Histopathology and immunohistochemistry

Among the specimens confirmed to be infected with clade 2.3.4.4b H5N1 HPAI virus, necropsies were conducted on a hooded crane (sample no. 22WC-042) collected on 17 November 2022 (Supplementary Figure S1). Organs including trachea, liver, spleen, kidney, pancreas, and cecal tonsils were fixed in 4% paraformaldehyde, embedded in paraffin, and sectioned into 5 μm-thick slices. These sections were subsequently stained with hematoxylin and eosin (H&E) for histopathological analysis and evaluated according to Landmann et al. (16). Immunohistochemical staining was carried out to detect the presence of the influenza virus antigen. The tissue sections were first incubated overnight at 4°C with goat anti-influenza A virus antibody (25 μg/ml, Invitrogen). After the overnight incubation, the sections were then incubated for 2 h at room temperature with biotinylated horse anti-goat IgG antibody (Vector Laboratories, Inc., USA). This was followed by incubation with horseradish peroxidase-conjugated streptavidin (Vector Laboratories, Inc.) for 1 h at room temperature. The positive signal indicating the presence of the virus was visualized using either diaminobenzidine (DAB) or Vector Red1 (Vector Laboratories, Inc.) as a substrate, and the sections were counterstained with methyl green for better contrast.

## 3 Descriptive results

Out of 221 dead hooded cranes, 194 tested positive for clade 2.3.4.4b H5N1 HPAI, and NGS was performed on 15 of the isolates. ML phylogenies analysis of the eight genes showed that all genes of H5N1 HPAI viruses isolated from hooded cranes in Korea and Japan clustered together and showed high sequence identities, indicating their close genetic relatedness (Supplementary Figure S2). Genotype of H5N1 HPAI viruses were analyzed according to the genotypic criteria established in a previous study (11). All viruses were classified as Kor22-23C genotype, except the A/hooded crane/Korea/22WC046-2/2022 virus which was Kor22-23B genotype, indicating that Kor22-23C was the dominant genotype among the hooded cranes (Table 1). The A/Hooded crane/Korea/WC042/2022 (H5N1) virus [22WC-042], isolated from the necropsied individuals, also showed 100% nucleotide sequence identity with the virus isolated from hooded cranes in Kagoshima, Japan, according to BLAST results (Supplementary Table S1), suggesting virus transmission between Korea and Japan.

**Table 1:** Genotypes of clade 2.3.4.4b H5N1 highly pathogenic avian influenza viruses isolated from the hooded crane carcasses in Suncheon Bay, Korea.

**No**.	**Virus**	Genotype^*^	**PB2**	**PB1**	**PA**	**HA**	**NP**	**NA**	**M**	**NS**
1	A/hooded crane/Korea/22WC026/2022	Kor22-23C	c	c	c	a	c	a	a	c
2	A/hooded crane/Korea/22WC042-1P/2022	Kor22-23C	c	c	c	a	c	a	a	c
3	A/hooded crane/Korea/22WC046-2/2022	Kor22-23B	b	b	b	b	b	b	b	b
4	A/hooded crane/Korea/22WC064/2022	Kor22-23C	c	c	c	a	c	a	a	c
5	A/hooded crane/Korea/22WC073/2022	Kor22-23C	c	c	c	a	c	a	a	c
6	A/hooded crane/Korea/22WC083/2022	Kor22-23C	c	c	c	a	c	a	a	c
7	A/hooded crane/Korea/22WC109/2022	Kor22-23C	c	c	c	a	c	a	a	c
8	A/hooded crane/Korea/22WC154/2022	Kor22-23C	c	c	c	a	c	a	a	c
9	A/hooded crane/Korea/22WC167/2022	Kor22-23C	c	c	c	a	c	a	a	c
10	A/hooded crane/Korea/22WC177/2022	Kor22-23C	c	c	c	a	c	a	a	c
11	A/hooded crane/Korea/22WC196/2022	Kor22-23C	c	c	c	a	c	a	a	c
12	A/hooded crane/Korea/22WC208/2022	Kor22-23C	c	c	c	a	c	a	a	c
13	A/hooded crane/Korea/22WC211/2022	Kor22-23C	c	c	c	a	c	a	a	c
14	A/hooded crane/Korea/22WC215/2022	Kor22-23C	c	c	c	a	c	a	a	c
15	A/hooded crane/Korea/22WC235/2022	Kor22-23C	c	c	c	a	c	a	a	c

PB2, polymerase basic protein1; PB1, polymerase basic protein 2; PA, polymerase acidic protein; HA, hemagglutinin; NP, nucleoprotein; NA, neuraminidase; MP, matrix protein; NS, non-structural protein.

^*^Genotyping according to Seo et al. (11).

On November 1, 2022, the first death of a hooded crane due to clade 2.3.4.4b H5N1 HPAI virus was reported in the Izumi Plain in Japan. Subsequently, on November 13, 2022, the first death of a hooded crane caused by the same virus strain was reported in Suncheon Bay, South Korea. Both Suncheon Bay and Izumi Plain, where the deaths were reported, are known as major wintering sites for hooded cranes in East Asia and lie along the same migratory route (Supplementary Figure S1) (17, 18). Given that at least 3,000 hooded cranes were confirmed to have migrated between Japan and Korea during November 2022 (19), it is highly likely that the virus was introduced during migration of hooded crane population. Taking into account the mass mortality of 1,476 hooded cranes in the Izumi Plain during the same period, it represents the death of 10% of the total hooded crane population (20).

**Figure 1:**
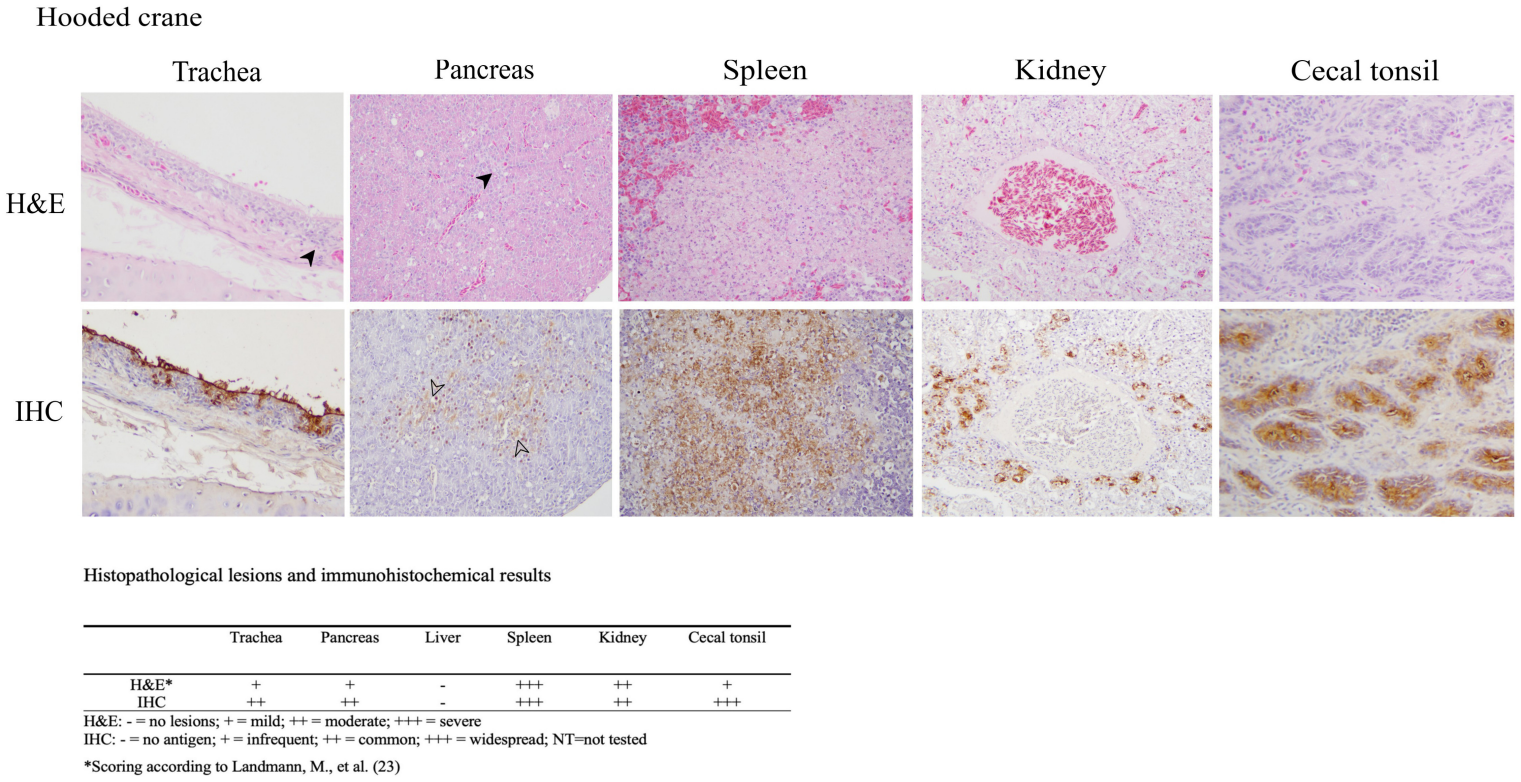
Results of H&E staining and immunohistochemical (IHC) testing in tissues of hooded crane (*Grus monacha*). Virus staining is the brown coloration. Trachea (magnification x200); infiltration of inflammatory cells (arrowhead), viral antigen in epithelial cells. Pancreas (magnification x200); inflammation, vacuolar degeneration (arrowhead). Spleen (magnification x400); necrotic foci with strong immunostaining positivity. Kidney (magnification x200); nuclear fragmentation and condensed nuclei in necrostic tubular epithelium with viral antigen in necrotic epithelium. Cecal tonsil (magnification x200); lymphoid depletion and viral antigen in the affected area. The lesion and immunostaining positivity scores are summarized under the images.

The histopathologic and immunohistochemical findings were consistent with previously reported HPAI virus infections in aquatic birds including multiple organs exhibiting necrotic and inflammatory lesions with corresponding positive immunostaining for influenza A virus within these areas, which indicates systemic infection (Figure 1) (21, 22). The trachea showed degeneration and necrosis of epithelial cells, accompanied by the infiltration of inflammatory cells such as lymphocytes and macrophages. Immunostaining was positive in some epithelial cells, including the brush border. In the pancreas, localized inflammatory cell infiltration, and vacuolar degeneration of acinar glandular epithelium was observed with immunostaining positivity acinar epithelium. The spleen showed focal hemorrhage, lymphoid depletion, and multifocal necrotic foci. Nuclear fragmentation and ghost nuclei were observed within the necrotic foci, with strong immunostaining. In the kidney, necrosis of tubules was observed with immunostaining of epithelial cells of the affected tubules. In the cecal tonsil, lymphoid depletion was observed in the region of the germinal center and immunostaining was strongly positive in the affected area. No lesions or immunostaining were identified in the liver. A limitation of our histopathology data is the exclusion of brain tissue, which precludes the evidence of potential neurotropism.

## 4 Conclusion

As the evolution and spread of clade 2.3.4.4b H5Nx HPAI virus continues, the host range and virulence are continuously changing. Unlike *Anseriformes* and *Charadriiforms*, hooded crane is not a previously reported natural host of influenza A virus (23), but we found mass die-off of this species and evidence of systemic infection. The genetic and pathological data established in this study would be useful as reference data for genomic surveillance and pathobiological study of HPAI viruses. In addition, hooded cranes are designated as a vulnerable species by the International Union for the Conservation of Nature (IUCN), raising concern not only for wide spread of HPAI but also for wildlife conservation. There is a need for a cross-border cooperation effort on active surveillance of HPAI in wild birds to monitor the evolution and spread of HPAI.

## Data Availability

The datasets presented in this study can be found in online repositories. The names of the repository/repositories and accession number(s) can be found in the article/Supplementary material.

## Supplementary material

Supplementary Figure 1 The geographical coordinates (latitude and longitude) of the sample collection sites. Suncheon Bay and Izumi Plain are wintering sites for hooded cranes and are located on the same migration route.

Supplementary Figure 2 Maximumlikelihood tree of (a) PB2, (b) PB1, (c) PA, (d) HA, (e) NP, (f) NA, (g) M, and (h) NS genes. Each color range represents different internal gene types. Viruses from hooded cranes in Korea are marked with blue dots, while those from hooded cranes in Japan are marked with red dots.
