# Supplementary figures and images for: Genetic and pathological analysis of hooded cranes (Grus monacha) naturally infected with clade 2.3.4.4b highly pathogenic avian influenza H5N1 virus in South Korea in the winter of 2022
Source: Front Vet Sci. 2024 Nov 6;11:1499440. doi: 10.3389/fvets.2024.1499440 (PMC11576466; doi:10.3389/fvets.2024.1499440)

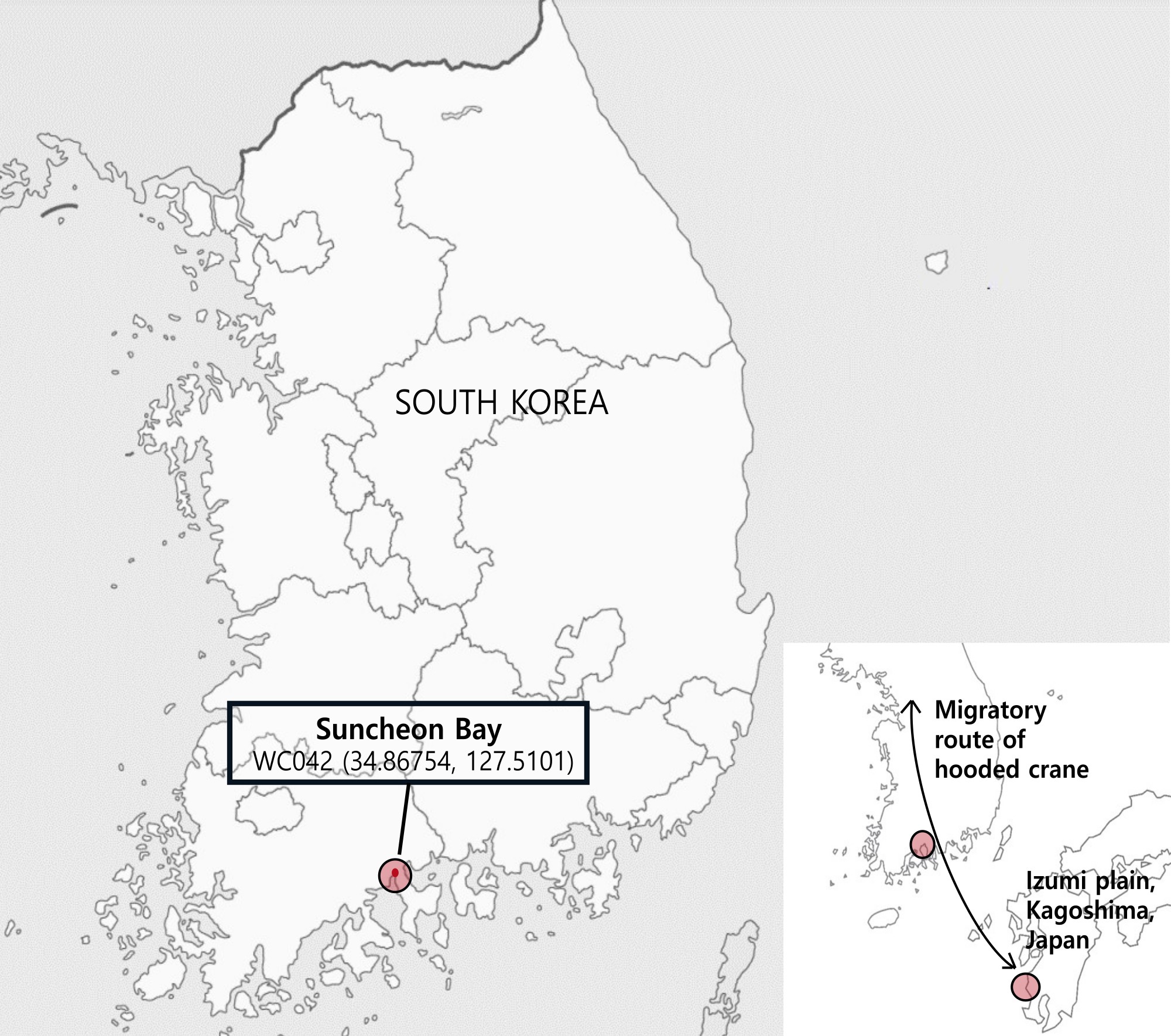

Supplement: Supplementary Figure 1 — The geographical coordinates (latitude and longitude) of the sample collection sites. Suncheon Bay and Izumi Plain are wintering sites for hooded cranes and are located on the same migration route. [file Image_1.jpeg]
