# Supplementary material for: Genetic and pathological analysis of hooded cranes (Grus monacha) naturally infected with clade 2.3.4.4b highly pathogenic avian influenza H5N1 virus in South Korea in the winter of 2022
Source: Front Vet Sci. 2024 Nov 6;11:1499440. doi: 10.3389/fvets.2024.1499440 (PMC11576466; doi:10.3389/fvets.2024.1499440)
Supplement: Supplementary Figure 2 — Maximumlikelihood tree of (a) PB2, (b) PB1, (c) PA, (d) HA, (e) NP, (f) NA, (g) M, and (h) NS genes. Each color range represents different internal gene types. Viruses from hooded cranes in Korea are marked with blue dots, while those from hooded cranes in Japan are marked with red dots. [file Image_2.pdf]

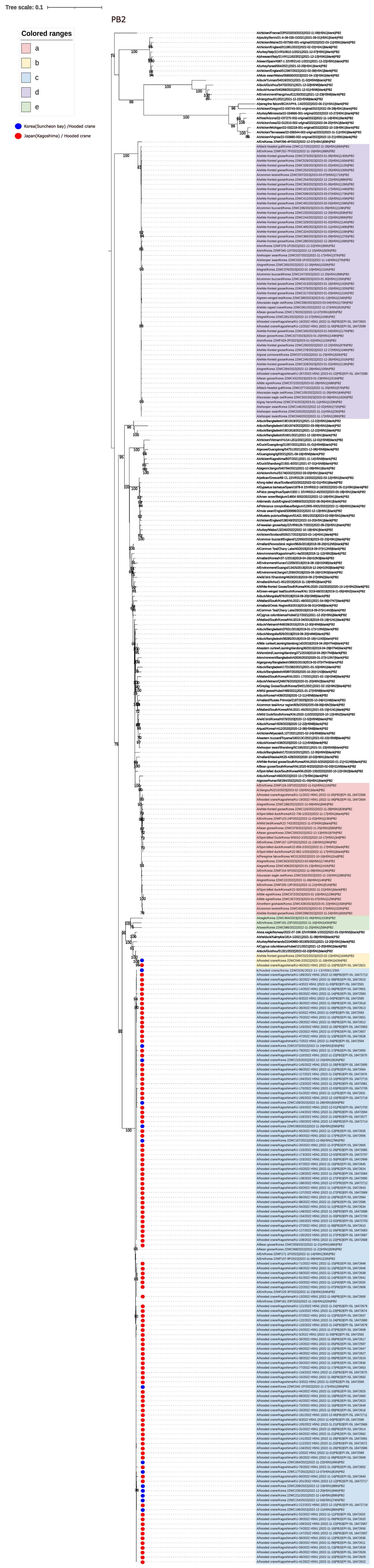
Tree scale: 0.1
PB2
Colored ranges
a
b
c
d
e
Korea(Suncheon bay) /Hooded crane
Japan(Kagoshima) / Hooded crane

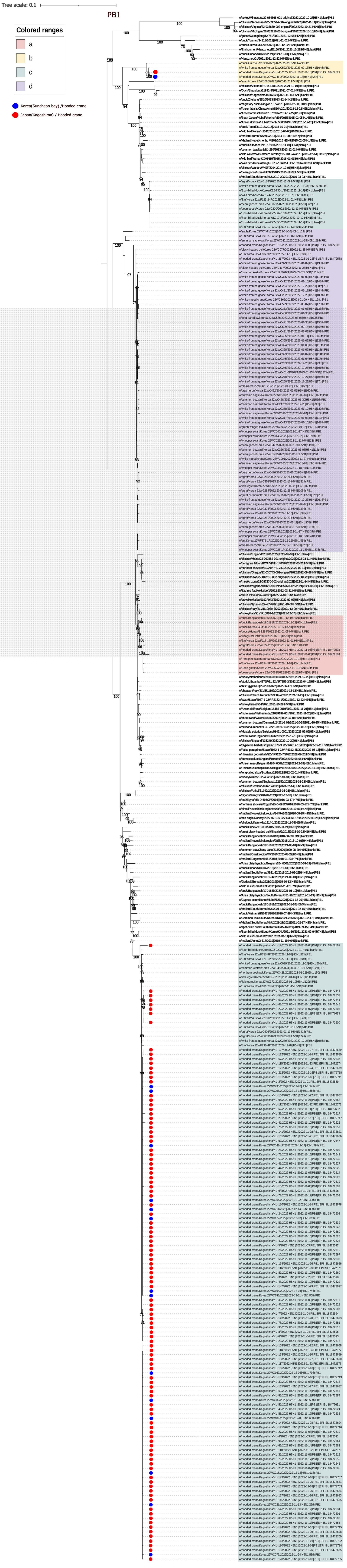
Tree scale: 0.1
PB1
Colored ranges
a
b
c
d
Korea(Suncheon bay) /Hooded crane
Japan(Kagoshima) / Hooded crane

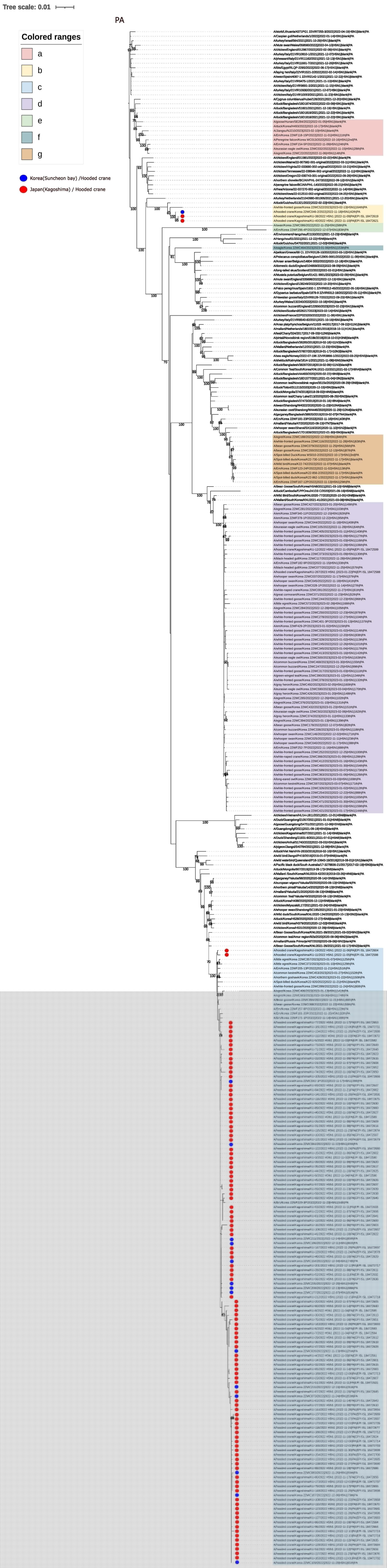
Tree scale: 0.01
PA
Colored ranges
a
b
c
d
e
f
g
Korea(Suncheon bay) /Hooded crane
Japan(Kagoshima) / Hooded crane

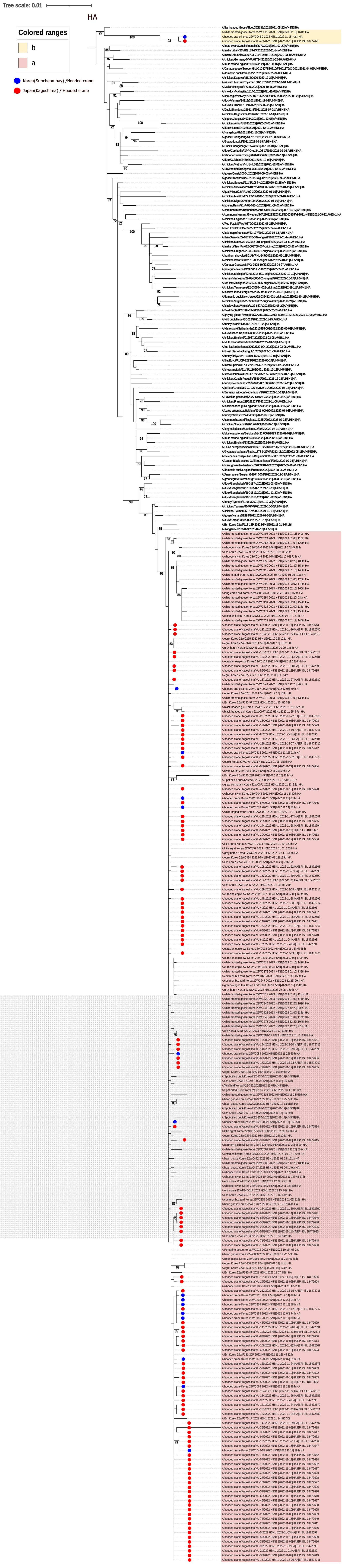
Tree scale: 0.01
HA
Colored ranges
b
a
Korea(Suncheon bay) /Hooded crane
Japan(Kagoshima) / Hooded crane

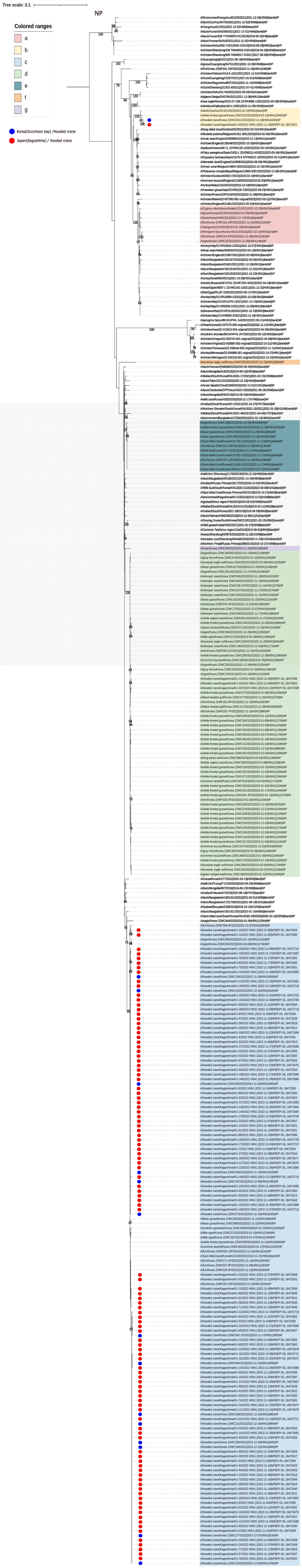
Tree scale: 0.1
NP
Colored ranges
a
b
c
d
e
f
g
Korea(Suncheon bay) /Hooded crane
Japan(Kagoshima) / Hooded crane

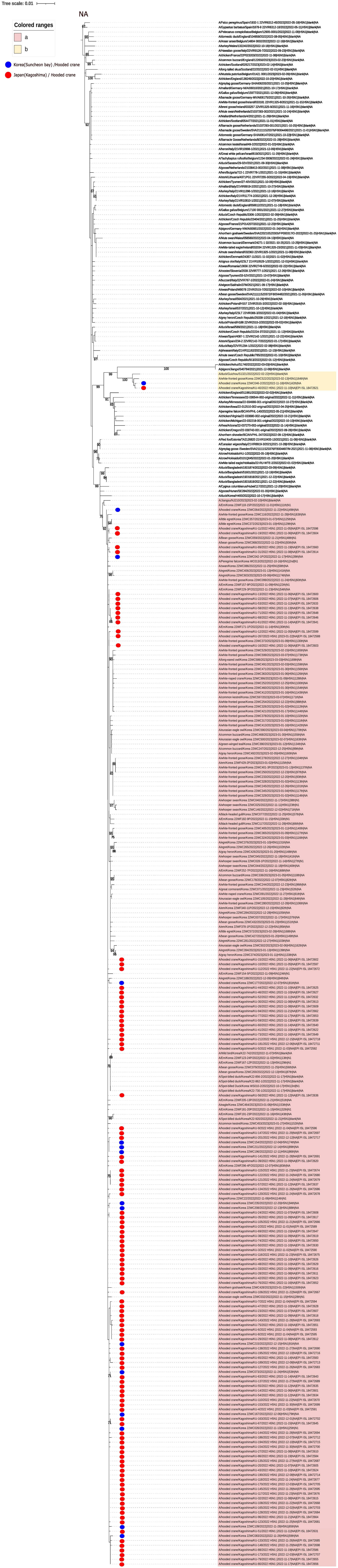
Tree scale: 0.01
NA
Colored ranges
a
b
Korea(Suncheon bay) /Hooded crane
Japan(Kagoshima) / Hooded crane

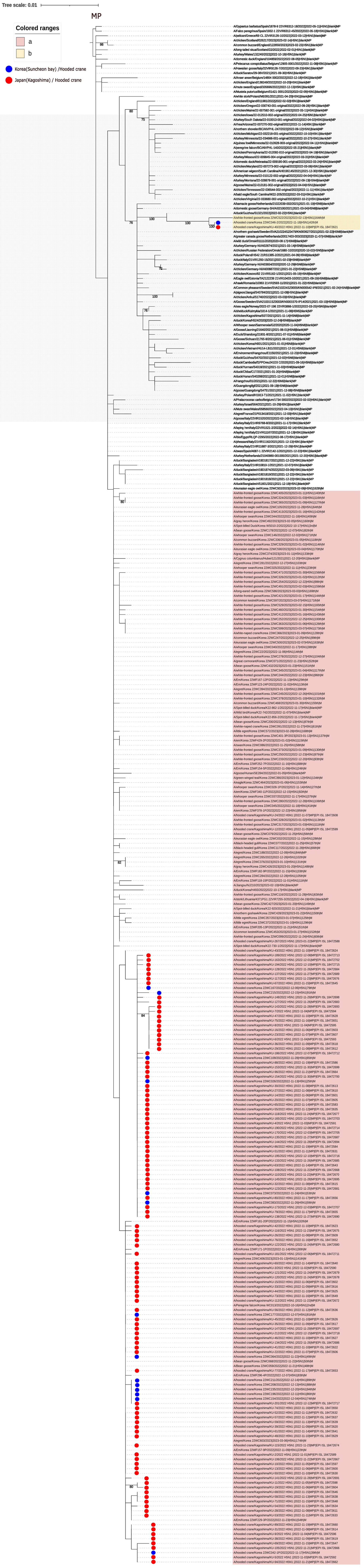

Tree scale: 0.01
MP
Colored ranges
a
b
Korea(Suncheon bay) /Hooded crane
Japan(Kagoshima) / Hooded crane
86
80

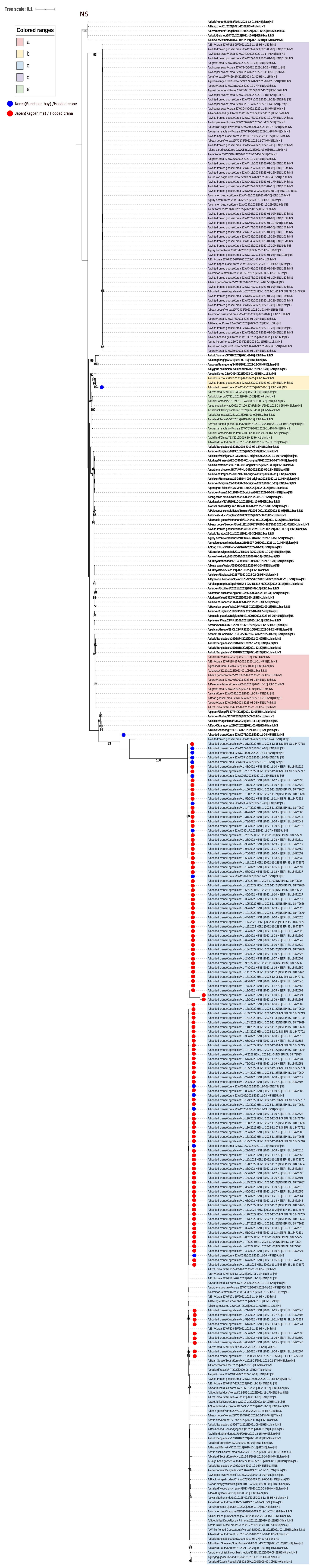
Tree scale: 0.1
NS
Colored ranges
a
b
c
d
e
Korea(Suncheon bay) /Hooded crane
Japan(Kagoshima) / Hooded crane
